# Diabetes education for better personalized management in pediatric patients

**DOI:** 10.1186/s12916-022-02709-2

**Published:** 2023-01-24

**Authors:** BMC Medicine

**Affiliations:** The Campus, 4 Crinan Street, London, UK

There are an estimated 1.1 million pediatric patients with type 1 diabetes (T1D) worldwide, and the once rare childhood type 2 diabetes (T2D) is dangerously on the rise. This developing issue has led to growing worries about the short and long term complications of the disease, as well as negative impacts on the quality of life of the patients and their families and the burden on healthcare systems. Despite various preventative and therapeutic approaches taken by governments and healthcare bodies to tackle diabetes, the rise in cases and widening inequalities in the management of the disease persist, indicating that not enough is being done. As the world ploughs through possible alternatives to tackle the issue of childhood diabetes, more attention is being turned towards facilitating knowledge and understanding of pediatric patients about their disease and its impact on the outcome. With all of this in mind, the question remains, how effectively can pediatric patients with diabetes be educated about their disease and treatment regimens to promote better management considering their developing cognitions?

Diabetes is a disease where blood glucose levels are too high. T1D is an autoimmune disease by which the body has diminished or no ability to produce the hormone insulin, whilst T2D is associated with poor lifestyle factors and occurs when the body ceases to produce enough insulin or cells develop resistance to the hormone. Diabetes can have detrimental consequences for both patients and their families. These include psychological impact stemming from emotional pressures, treatment complexity, financial costs and reaction of the community (particularly peer groups) towards the patients, as well as short and long-term complications caused by the disease. Some young people view diabetes as an impediment to achieving their personal and academic goals due to the struggles they and their families face in living with diabetes. To make matters even more concerning, there are widening inequalities in pediatric diabetes care that can potentially result in poorer outcomes in patients from ethnic minorities and low socioeconomic backgrounds.

Perhaps one way of tackling this mounting problem is by increasing the understanding and acceptance of pediatric diabetes patients of their disease through education, which could also improve patient involvement and experience. Such education is of vital importance for both physical and mental health of the patients. This is since comprehending the cause of diabetes, in addition to the consequences of poor management and adjusting to self-care routines can be challenging to young people of different ages. It is, therefore, no surprise that healthcare professionals are turning their attention towards educational interventions in such patients in a bid to bring the situation under better control.

To improve pediatric patient cooperation, educational interventions need to take the fundamental principles of child learning into consideration. While, particular attention has been given to studying the effectiveness of such educational programmes in pediatric patients with T1D, there is no current data on T2D. Although, results from numerous studies on educational methods have shown significant improvements in HbA1c levels and treatment regimen perceptions in 9-11 year-old children with T1D management difficulties, the opposite has been shown in studies conducted on adolescent patients. For example, the CASCADE trial concluded that the structured education intervention did not improve HbA1c levels in 13-19 year-old T1D patients with poor control, and its delivery was met with major challenges which was also confirmed by the CHOICE trial. These challenges can be attributed to poor literacy and numeracy skills, inadequate parental involvement, the nature of complex intervention strategies and lack of engagement by the participants for different reasons.

Despite current challenges in educational interventions for pediatric diabetes patients, they can, in principle, be multipurpose by promoting management and prevention of the disease as well as breaking social stigma. A recent study by Machado Mourao *et al*. 2022 delved into assessing theatre play, games, educational package, and workshop training as the intervention for raising knowledge and awareness about diabetes in staff and 7-12 year-old, mostly non-diabetic children within a school environment. They observed positive changes in the understanding and perception of students and staff particularly in relation to hypoglycaemia and sugar intake by diabetes sufferers. The, at times, conflicting yet promising findings from the above trials demonstrate that educational intervention can be an effective management tool if it is tailored to meet the needs of different pediatric groups and overcome challenges encountered in previous studies.

Charities like Diabetes UK are leading the way on this front by introducing relatable storytelling guides for children with T1D. Playful educational apps and videos can also be useful and engaging platforms for children and young people with diabetes. Whilst, the UK government and NHS currently offer numerous T2D prevention as well as T1D/ T2D management programmes, none are targeted at pediatric patients since more evidence is required on the effectiveness of such interventions in this patient group.

The 4T (Teamwork, Targets, Technology, and Tight Control) clinical trial study is examining whether implementing a combination of proven methods and emerging diabetes technology into clinical practice can reduce HbA1c and T1D burden as well as improve well-being in newly diagnosed pediatric patients with T1D. Similarly, another interesting trial (GET-IT-T1D) is investigating whether group education visits can result in better HbA1c, less adverse outcomes and better psychosocial outcomes in comparison to usual care in patients transitioning from pediatric to adult diabetes care. Such studies can shed light on how compliant diabetes patients can remain as emerging adults.

As we recently marked World Diabetes Day, it is imperative to express that education holds great potential for pediatric patients with diabetes and their families. It empowers them with diabetes self-management skills and gently guides them towards approaching living with the disease and adhering to relevant treatments more positively. With rising T2D cases, providing such education to pediatric patients with obesity could act as a diabetes prevention tool in this group and also help to build rapport between patients and their clinical care team. A well-structured and individualized educational intervention goes hand in hand with a range of already available government and charity led programmes and strategies, which may be the stepping stone to prevention and better management of diabetes in children and adolescents.

Investment of funding on large scale and well-designed educational intervention trials will be essential. This will not only help to gather the evidence needed on the benefits of these programmes, but can also reduce inequalities by enabling the participation of pediatric patients from ethnic minorities and deprived areas.

